# Medicare Support for Dental and Podiatry Graduate Medical Education Programs

**DOI:** 10.1001/jamanetworkopen.2021.11797

**Published:** 2021-05-27

**Authors:** Candice Chen, YoonKyung Chung, Geoffrey Broadbent, Elizabeth Mertz

**Affiliations:** 1 Department of Health Policy and Management, Fitzhugh Mullan Institute for Health Workforce Equity, Milken Institute School of Public Health, George Washington University, Washington, District of Columbia; 2 Robert Graham Center, Washington, District of Columbia; 3 School of Medicine and Health Sciences, George Washington School of Medicine, Washington, DC; 4 School of Dentistry, University of California, San Francisco

## Abstract

**Importance:**

Oral health care faces ongoing workforce challenges that affect patient access and outcomes. While the Medicare program provides an estimated $14.6 billion annually in graduate medical education (GME) payments to teaching hospitals, including explicit support for dental and podiatry programs, little is known about the level or distribution of this public investment in the oral health and podiatry workforce.

**Objective:**

To examine Medicare GME payments to teaching hospitals for dental and podiatry residents from 1998 to 2018, as well as the distribution of federal support among states, territories, and the District of Columbia.

**Design, Setting, and Participants:**

This cross-sectional study was conducted using data from 1252 US teaching hospitals. Data were analyzed from May through August 2020.

**Exposures:**

Dental and podiatry residency training.

**Main Outcomes and Measures:**

Medicare dental and podiatry GME payments were examined.

**Results:**

Among 1252 teaching hospitals, Medicare provided nearly $730 million in dental and podiatry GME payments in 2018. From 1998 to 2018, the number of residents supported more than doubled, increasing from 2340 residents to 4856 residents, for a 2.1-fold increase, while Medicare payments for dental and podiatry GME increased from $279 950 531 to $729 277 090, for a 2.6-fold increase. In 2018, an estimated 3504 of 4856 supported positions (72.2%) were dental. Medicare GME payments varied widely among states, territories, and the District of Columbia, with per capita payments by state, territory, and district population ranging from $0.05 in Puerto Rico to $14.24 in New York, while 6 states received no support for dental or podiatry residency programs.

**Conclusions and Relevance:**

These findings suggest that dental and podiatry GME represents a substantial public investment, and deliberate policy decisions are needed to target this nearly $730 million and growing investment to address the nation’s priority oral and podiatry health needs.

## Key Points

**Question:**

How much does the US invest in training the dental and podiatry workforce?

**Findings:**

In this cross-sectional study of US teaching hospitals, Medicare provided nearly $730 million in dental and podiatry graduate medical education (GME) payments to teaching hospitals in 2018, with per capita payments varying across states, territories, and the District of Columbia. Medicare payments for dental and podiatry GME increased from 1998 to 2018.

**Meaning:**

These findings suggest that dental and podiatry GME represents a substantial, growing public investment, and deliberate policy reform may be warranted to address national oral and podiatric health needs.

## Teaser

This cross-sectional study investigates the amount of Medicare funding for dental and podiatry graduate medical education programs at teaching hospitals from 1998 to 2018.

## Introduction

Graduate medical education (GME), or residency or fellowship training, represents the largest public investment in health workforce development in the US. The federal government and the governments of states, territories, and the District of Columbia provide more than $16 billion to support GME training programs annually.^1^ In 2018, the Medicare program alone provided $14.6 billion in GME payments to teaching hospitals.^2^ Research on the physician workforce has found that GME is associated with the overall number, specialty and geographic distribution, and practice patterns of physicians,^3,4,5,6,7,8^ all of which have implications for health care access, quality, and cost.^9,10,11^ However, despite this public investment in GME, the US continues to struggle with a maldistribution of physicians in high-need specialties, such as primary care and behavioral health, and in rural and underserved areas.^12,13^

Research into GME has largely focused on the physician workforce. However, Medicare explicitly funds dental and podiatry residency, although publicly available data on this support is available only in aggregate (ie, dental and podiatry positions and funds cannot be separated). Dental and podiatry programs are also exempt from the 1997 Balanced Budget Act cap on the number of GME positions eligible for Medicare support at each hospital. As a result, hospitals are able to increase dental and podiatry GME positions and receive additional Medicare GME funding without the limits of resident caps, while physician residency positions are subject to these limits. Despite these public investments, oral health care and podiatry have faced ongoing workforce and access challenges. An estimated 60 million people live in dental health professional shortage areas,^14^ and the Health Resources and Services Administration (HRSA) projects that all 50 states and the District of Columbia will experience a shortage of dentists in 2025.^15^ The agency further projects a shortage of more than 4000 podiatrists by 2030,^16^ although there is no equivalent shortage area analysis for the podiatry workforce.

In contrast to Medicare-supported GME, HRSA support for oral health training programs was funded at $40.67 million in fiscal year 2020. The HRSA programs provide grants to support training in dental and dental hygiene schools, dental residency programs, and a State Oral Health Workforce Improvement Program. In academic year 2018 to 2019, HRSA supported the training of 494 dental residents.^17^ The agency provides no explicit support for podiatry training.

Dental and podiatry residency programs have unique characteristics. In podiatry, a 3-year, largely hospital-based or academic health center–based residency is standard after graduation. In dentistry, residency training is not universally required. Only 2 states (New York and Delaware) require residency training for a dentist to be licensed. In contrast, nearly all states require podiatry residency training for licensing,^18^ and every state requires physicians to complete some GME training in the US to be licensed.^19^ The unique characteristics of dental residency programs are also likely to influence the level of federal support for different dental specialties. Medicare GME formulas largely tie GME payments to hospitals. By statute, Medicare provides 2 types of payments: direct and indirect GME. Indirect GME is an adjustment to the inpatient prospective payment system, meaning only organizations billing inpatient services will qualify for indirect GME payments. Indirect GME generally comprises the larger of the 2 payments, making up nearly 75% of total payments.^20^ While outpatient organizations can qualify for direct GME, their payments would be limited, given that direct GME calculations are based on Medicare patient share, which tends to be lower in outpatient vs inpatient settings. Oral health–focused organizations may have little to no Medicare share owing to limited coverage of oral health services by the Medicare program.^21^

Among 12 recognized specialties in dentistry, program duration ranges from 1 to 6 years, with varying intensity of training time spent in a hospital setting. For example, oral and maxillofacial surgery is heavily hospital based. However, advanced education in general dentistry and general practice residency programs, which made up 35% of 773 dental residency programs and 53% of graduates in 2019,^22^ include community-based programs, hospital-based programs, and dental school–based programs. Orthodontics, endodontics, periodontics, and prosthodontics programs are completed primarily in nonhospital clinical settings, and dental public health is a nonclinical specialty, making programs in this specialty much less likely to receive Medicare GME support.

Very little is known about the distribution of Medicare GME support specifically for dental and podiatry residency programs. This study examined Medicare GME payments for dental and podiatry residency programs at teaching hospitals from 1998 to 2018, as well as the distribution of these payments across states, territories, and the District of Columbia.

## Methods

The George Washington University did not consider this cross-sectional study to be human participants research given that data were not collected through intervention or interaction with individuals and no private or identifiable individual information was used; the university therefore determined that institutional review board submission and informed consent were not required. This study is reported following the Strengthening the Reporting of Observational Studies in Epidemiology (STROBE) reporting guideline.

We used fiscal years 1998 to 2018 Medicare hospital cost reports from the Centers for Medicare & Medicaid Services (CMS) Healthcare Cost Report Information System to identify teaching hospitals reporting dental and podiatry residency positions. Medicare hospital cost reports are publicly available, organization-level administrative data sets. Teaching hospitals report the number of combined dental and podiatry residents, number of physician residents trained, physician resident cap, and total payments for direct and indirect GME.

### Statistical Analysis

We calculated the annual total direct and indirect dental and podiatry resident full-time equivalents (FTE) supported by Medicare. The program provides 2 types of GME payments (ie, direct and indirect), and resident FTE is determined separately for each payment. To calculate the direct, indirect, and total (ie, direct plus indirect) dental and podiatry GME payments, we calculated a direct and indirect per resident GME payment for each hospital: the hospital’s total direct or indirect GME payments divided by the sum of the number of physician residents trained (which is subject to the physician resident cap) and the number of dental and podiatry residents. Hospital-level dental and podiatry GME payments were then calculated by multiplying the number of direct and indirect dental and podiatry residents by the corresponding GME payment rates; these payments were then aggregated to determine total annual Medicare GME payments for dental and podiatry positions. We excluded teaching hospitals in the bottom or top 1% of GME payment rate, as well as those with missing values for FTEs. All statistical analyses were descriptive and were conducted using Stata statistical software version 16.1 (StataCorp) from May through August 2020. Reporting of *P* values is not applicable given that realized Medicare GME payments were described over time and no additional statistical tests were performed. All dollar amounts were inflation-adjusted to 2018 using the US Consumer Price Index for all urban consumers.

We examined the state distribution of Medicare dental and podiatry GME support in 2018, calculating the number of residents, amount of GME payments per population, and state mean GME payment per resident. Population data are from 2018 US Census Bureau estimates. We also examined dental vs podiatry residency positions using the publicly available Central Application Service for Podiatric Residencies directory,^23^ which provides affiliated institutions and the number of approved resident positions for podiatry residency programs. Affiliated institutions were matched by location and name to Medicare hospital names to determine the maximum number of Medicare-supported podiatry residents in teaching hospitals. Dental resident numbers were identified as any remaining Medicare-supported resident positions in the pooled dental and podiatry positions. The American Dental Education Association provides similar residency program–level information on program length and available positions; however, it does not report hospital affiliations needed to match to the Medicare hospital cost reports.

## Results

**Figure 1.**
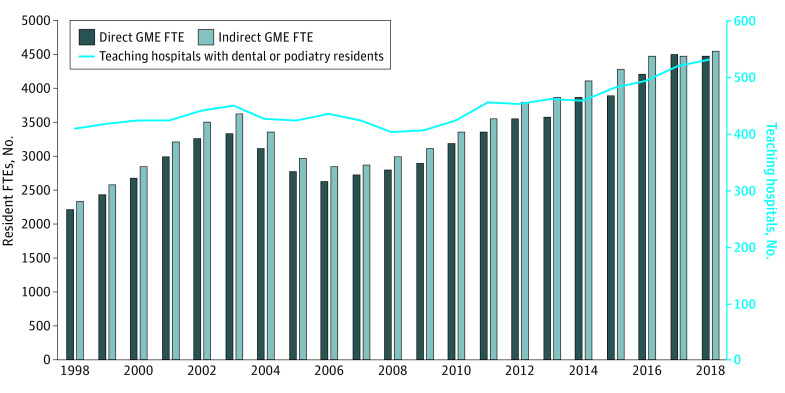
Number of Medicare Dental and Podiatry Resident Full-Time Equivalents (FTEs) and Teaching Hospitals GME indicates graduate medical education.

**Figure 2.**
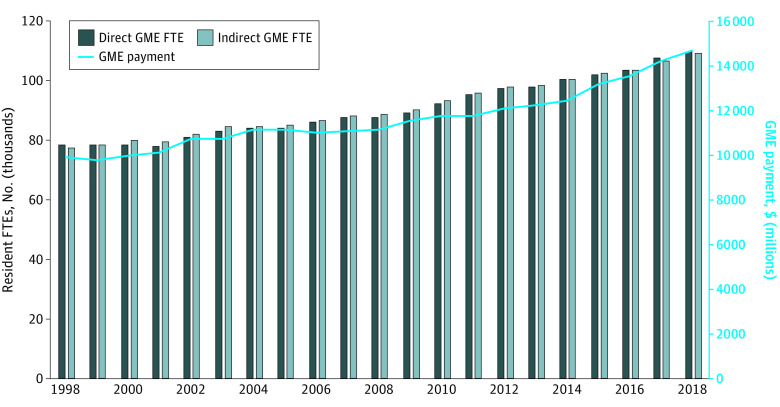
Number of Medicare Graduate Medical Education (GME) Resident Full-Time Equivalents (FTEs) and Payment Amounts Values are given in 2018 US dollars. All dollar amounts were inflation-adjusted to 2018 using the US Consumer Price Index for all urban consumers.

From 1998 to 2018, the number of teaching hospitals with dental and podiatry residency programs increased from 409 to 532 hospitals, while the overall number of teaching hospitals with physician GME programs did not increase, with 1252 teaching hospitals in 1998 and 1206 teaching hospitals in 2018. The total number of dental and podiatry residents over the study period initially increased until 2003, decreased until 2006, and then steadily increased in subsequent years (Figure 1). In 2018, Medicare supported 4856 dental and podiatry GME positions, compared with 2340 such positions in 1998, for a 2.1-fold increase. The overall number of Medicare-supported residents experienced steadier, slower growth during the same period, increasing from 78 178 positions in 1998 to 109 395 positions in 2018, for a 40.0% increase (Figure 2). In 2018, an estimated 1352 dental and podiatry positions (27.8%) were podiatry positions and 3504 positions (72.2%) were dental positions.

**Figure 3.**
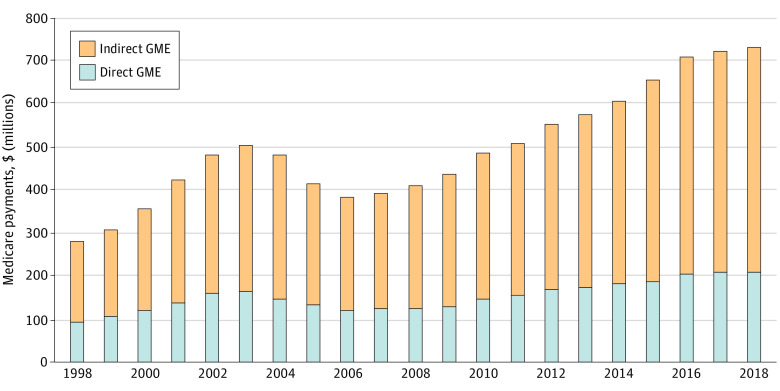
Medicare Direct and Indirect Dental and Podiatry Graduate Medical Education (GME) Payments Values are given in 2018 US dollars. All dollar amounts were inflation-adjusted to 2018 using the US Consumer Price Index for all urban consumers.

Total Medicare dental and podiatry GME payments followed similar trends to the number of residents, increasing from $279 950 531 in 1998 to $502 344,851 in 2003, decreasing to $382 605 584 in 2006, and then steadily increasing to $729 277 090 in 2018 (Figure 3). There was a 2.6-fold increase over the study period.

**Figure 4.**
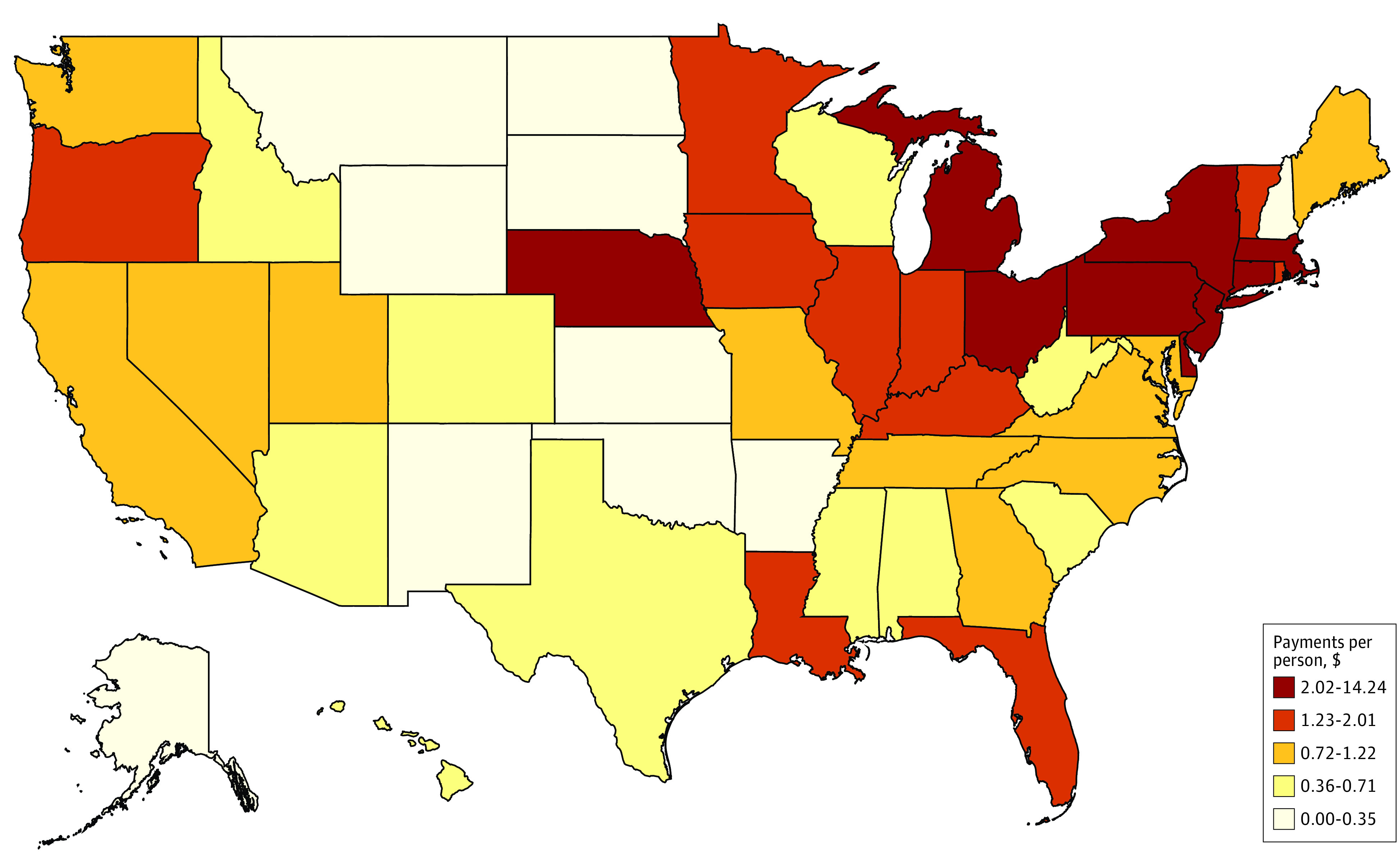
Per Capita 2018 Dental and Podiatry Medicare Graduate Medical Education (GME) Payments Payments are per person by state, territory, or district population.

In 2018, Medicare support for dental and podiatry residency programs varied across states, territories, and the District of Columbia (Figure 4). While 6 states (Alaska, Montana, North Dakota, New Hampshire, South Dakota, and Wyoming) saw no dental or podiatry GME support, the remaining states and territories and the District of Columbia received total dental and podiatry Medicare GME payments ranging from $159 245 in Puerto Rico to $278 million New York. Among states and territories with dental and podiatry residents and the District of Columbia, the number of such resident positions supported per 100 000 members of the population ranged from 0.12 resident positions in Puerto Rico to 8.3 resident positions in New York, and the GME payments per person by state, territory, or district population ranged from $0.05 in Puerto Rico to $14.24 in New York. The Table provides the top and bottom 10 states and territories and the District of Columbia by Medicare dental and podiatry GME payments per capita. Dental and podiatry Medicare GME support levels for all states are provided in the eTable in the Supplement.

**Table.** Top and Bottom 10 Medicare Dental and Podiatry GME Payments Per Capita by State, Territory, and District, 2018

State, territory, or district	FTEs		GME payment		
	Total, No.	No. per 100 000 population members	Total, $	Per capita, $	Per resident position, $
1. NY	1621.69	8.30	278 064 736	14.24	171 466
2. DC	47.72	6.80	6 722 461	9.58	140 873
3. CT	153.80	4.31	25 712 004	7.20	167 178
4. PA	378.99	2.96	61 712 368	4.82	162 834
5. NJ	204.95	2.31	32 842 874	3.70	160 248
6. MI	189.99	1.90	32 353 866	3.24	170 292
7. OH	265.66	2.28	37 563 144	3.22	141 396
8. DE	17.62	1.83	3 051 782	3.16	173 200
9. MA	90.30	1.31	15 268 321	2.22	169 084
10. NE	27.49	1.43	4 219 502	2.19	153 492
43. NM	7.96	0.38	667 668	0.32	83 878
44. OK	11.67	0.30	924 901	0.23	79 255
45. AR	4.65	0.15	657 434	0.22	141 384
46. PR	3.84	0.12	159 245	0.05	41 470
47. AK	0	0	0	0	0
48. MT	0	0	0	0	0
49. ND	0	0	0	0	0
50. NH	0	0	0	0	0
51. SD	0	0	0	0	0
52. WY	0	0	0	0	0
National	4856	1.49	729 297 267	2.23	150 190

Abbreviations: FTE, full-time equivalents; GME, graduate medical education.

## Discussion

This cross-sectional study found that Medicare funding of dental and podiatry GME was substantial and growing. In 2018, Medicare provided nearly $730 million in GME payments to teaching hospitals to support 4856 dental and podiatry residency positions. According to our calculations, more than 70% of the supported positions were in dental residency programs, despite limited support by the Medicare program for dental services. In contrast, HRSA’s oral health training programs were funded at $40.67 million and supported 494 dental residents in fiscal year 2020.^17^ The American Dental Association reported that overall enrollment in all dental residency programs was 7355 residents in 2019 to 2020, up from 6095 residents in 2009 to 2010.^20^

While the overall number of Medicare-supported residency positions increased nearly 40.0% over the 20-year study period, the number of dental and podiatry positions more than doubled from 1998 to 2018 and inflation-adjusted Medicare payments for dental and podiatry GME increased 2.6-fold. Because of the absence of a resident cap for dental and podiatry positions, hospitals with existing programs could expand and hospitals at their physician residency caps could still establish and receive Medicare GME payments for new dental and podiatry programs.

The Medicare Payment Advisory Commission and National Academy of Medicine have called for GME payment reforms to align training with the needs of high value health care.^24,25^ Dental and podiatry GME represents an important component of this public investment, and targeted reforms can address oral and podiatry workforce quality and distribution. The workforce for podiatry, a specialty that requires residency, may be particularly important in multidisciplinary team approaches for the care of patients with diabetes, among whom evidence has found that contact with podiatry can reduce the risk of lower extremity amputations.^26^

However, as residency training is not required for dentists as it is with physicians and podiatrists, the “if you build it, they will come” strategy may have different outcomes for this workforce compared with the physician workforce, in which evidence has demonstrated that future practice locations are associated with the location of GME.^4,27^ The residency requirement difference may also be a contributing factor associated with the state-by-state variation in dental and podiatry positions and related Medicare GME support, with 6 states receiving no dental or podiatry GME support, and the remaining states and territories and the District of Columbia receiving GME payments per person by state, territory, or district population that ranged from $0.05 to $14.24. However, interest in dental residency programs is strong. The American Dental Association reported a 20% increase in enrollments over the past decade, with particular growth in pediatric, general practice, and advance education in general practice residencies.^22^ In 2019, 11 341 individuals applied for general practice residency programs in dentistry, and 1113 individuals were enrolled.^22^ The growth of dental residency programs suggests that there is demand for this additional training, and new or expanded residency programs may be an important strategy for states, territories, and the District of Columbia to address oral health workforce needs.

One notable trend over the study period was the initial rise, then fall of resident positions from 1998 to 2006. The 1997 Balanced Budget Act allowed hospitals to include the time residents train in nonhospital settings in determining Medicare GME payments, ostensibly to encourage training in nonhospital, community-based settings. In 2003, CMS issued new guidelines regarding resident training in nonhospital settings, limiting payments to positions for which hospitals had incurred training costs since the inception of the program.^28^ This policy change may be associated with the decrease in positions seen from 2003 to 2006. However, teaching hospitals demonstrated recovery and continued growth in positions and payments after 2006.

Community-based training is an important strategy to increase underserved practice choices.^29,30^ New York University (NYU) Langone Health, notable for receiving more Medicare funding for dental and podiatry GME than any other teaching hospital, partners with clinical sites across the US, often basing dental residency programs in community and tribal health centers.^31^ Working within CMS policies and regulations, this program uses a targeted, community-based training strategy. However, NYU Langone Health is the exception, not the rule. Medicare policy directly couples GME payments to hospitals. The larger of 2 payments, indirect GME, is an add-on payment to inpatient reimbursements. Nonhospitals are not eligible to directly receive indirect GME payments. While supporting training time in nonhospital settings may allow some community-based training, the funding and decisions remain in the hands of the hospital, and residency programs that spend little to no time in the hospital setting remain disadvantaged. Furthermore, some dental residency programs charge tuition, particularly if no GME support exists. This tuition adds to the already substantial debt burden of future dentists and may discourage undergoing residency training in more community-based specialties or conducting practice in underserved settings.

In addressing oral health care and podiatry needs, better understanding and attention to the role of Medicare in developing the oral health and podiatry workforce is warranted. Medicare funding for dental and podiatry GME is substantial, and as with the physician workforce, Medicare investments in this workforce require measurement, transparency, and accountability. While the growth in dental and podiatry positions may reflect growth in training programs and enrollment in both disciplines, questions remain as to whether growth is needed and whether the focus on hospital-based specialties will address community-based needs. For example, growth in Medicare podiatry support may be associated with a change by the American Board of Podiatric Medicine to require 3 years of residency for certification,^32^ whereas podiatry residency programs previously ranged from 1 to 3 years.^33^ The absence of a resident cap for dental and podiatry programs would allow programs to increase their Medicare GME support in this situation; however, the question arises as to the value of this additional public support.

Given the relative size and growth of support, it is important to ask whether Medicare-funded programs are producing health care providers who are addressing the nation’s priority health care needs. Ongoing challenges with oral health access suggest that the answer is no, while more information is needed to assess supply and demand for podiatry services. Additional metrics and measurements are needed to track the outcomes of public GME funding, particularly examining whether growth in GME support is addressing dental health professional shortage areas and areas of podiatry workforce shortages over time.

### Limitations

This study has several limitations. Our analysis is limited to data submitted by teaching hospitals for Medicare administrative and payment purposes. Resident positions represent only FTEs eligible for payment, are not equal to individual residents, and do not represent the full public investment in dental and podiatry GME. In addition, dental and podiatry residents cannot be disaggregated in the data, and using the Podiatric Residencies directory to determine hospital podiatry vs dental positions may overestimate or underestimate positions in either profession.

## Conclusions

This study found that Medicare provided nearly $730 million in GME payments to teaching hospitals to support 4856 dental and podiatry residency positions in 2018. Medicare GME funding has been heavily debated and discussed since its inception, and this debate has intensified over the last decade, with calls for major GME reform. However, these discussions have often failed to consider dental and podiatry GME. The oral and podiatry health workforces are vital for the health and wellness of the US population, and Medicare GME investments are substantial. Measurement, transparency, and accountability are needed, as are deliberate policy decisions to ensure that this nearly $730 million and growing public investment is targeted to address the nation’s oral and podiatry health needs.

## Notes

Supplement. **eTable.** Dental and Podiatry Resident Full-Time Equivalents and Medicare Graduate Medical Education Support Amount by State, Territory, or District in Fiscal Year 2018
