# Supplementary material for: Medicare Support for Dental and Podiatry Graduate Medical Education Programs
Source: JAMA Netw Open. 2021 May 27;4(5):e2111797. doi: 10.1001/jamanetworkopen.2021.11797 (PMC8160593; doi:10.1001/jamanetworkopen.2021.11797)
Supplement: Supplement. — eTable. Dental and Podiatry Resident Full-Time Equivalents and Medicare Graduate Medical Education Support Amount by State, Territory, or District in Fiscal Year 2018 [file jamanetwopen-e2111797-s001.pdf]

Supplemental Online Content

Chen C, Chung Y, Broadbent G, Mertz E. Medicare support for dental and podiatry graduate medical education programs. *JAMA Netw Open*. 2021;4(5):e2111797.
doi:10.1001/jamanetworkopen.2021.11797

eTable. Dental and Podiatry Resident Full-Time Equivalents and Medicare Graduate Medical Education Support Amount by State, Territory, or District in Fiscal Year 2018

This supplemental material has been provided by the authors to give readers additional information about their work.

© 2021 Chen C et al. *JAMA Network Open.*

eTable. Dental and Podiatry Resident Full-Time Equivalents and Medicare Graduate Medical Education Support Amount by State, Territory, or District in Fiscal Year 2018

State	Dental/ Pod FTE*	Dental FTE	Podiatry FTE	Dental/Pod FTE per 100,000 Pop	Dental/Pod Total GME $, 1998 (2018 $)	Dental/Pod Total GME $, 2018	Dental/ Pod GME $ per Capita	Dental/Pod GME Payment per Resident
AK	0.00	0.00	0.00	0.00	0	0	0.00	0.00
AL	23.67	23.67	0.00	0.48	1,289,529	2,615,328	0.54	110,491
AR	4.65	4.65	0.00	0.15	0	657,434	0.22	141,384
AZ	22.98	14.31	8.67	0.32	116,336	3,566,851	0.50	155,215
CA	273.02	198.40	74.62	0.69	12,225,129	37,372,004	0.95	136,884
CO	38.48	25.13	13.35	0.68	1,152,814	2,453,243	0.43	63,754
CT	153.80	126.96	26.84	4.31	14,024,941	25,712,004	7.20	167,178
DC	47.72	23.72	24.00	6.80	4,387,122	6,722,461	9.58	140,873
DE	17.62	14.62	3.00	1.83	2,210,855	3,051,782	3.16	173,200
FL	253.20	169.36	83.84	1.19	13,688,899	32,582,298	1.53	128,682
GA	88.79	73.79	15.00	0.84	3,246,910	9,049,738	0.86	101,923
HI	3.21	3.21	0.00	0.23	68,630	614,626	0.43	191,472
IA	34.74	24.55	10.19	1.10	2,172,919	5,322,016	1.69	153,196
ID	8.00	8.00	0.00	0.46	0	1,214,422	0.69	151,803
IL	156.63	91.19	65.44	1.23	10,794,543	18,434,314	1.45	117,693
IN	61.00	31.19	29.81	0.91	3,068,956	8,808,980	1.32	144,410
KS	5.79	5.79	0.00	0.20	10,571	1,026,541	0.35	177,296
KY	46.89	31.89	15.00	1.05	1,273,792	5,633,101	1.26	120,134
LA	164.14	155.14	9.00	3.52	1,029,762	6,218,972	1.33	37,888
MA	90.30	48.30	42.00	1.31	6,583,718	15,300,000	2.22	169,084
MD	34.47	34.47	0.00	0.57	695,527	6,504,913	1.08	188,712
ME	8.38	8.38	0.00	0.63	0	1,472,568	1.10	175,724
MI	189.99	88.89	101.01	1.90	9,627,458	32,353,866	3.24	170,292
MN	54.81	41.75	13.06	0.98	2,473,617	8,210,040	1.46	149,791
MO	52.61	31.61	21.00	0.86	3,780,490	5,089,889	0.83	96,748
MS	23.97	23.97	0.00	0.80	454,348	2,119,405	0.71	88,419
MT	0.00	0.00	0.00	0.00	0	0	0.00	0.00
NC	70.91	61.91	9.00	0.68	6,040,518	12,135,768	1.17	171,143
ND	0.00	0.00	0.00	0.00	346,212	0	0.00	0.00
NE	27.49	27.49	0.00	1.43	3,046,289	4,219,502	2.19	153,492
NH	0.00	0.00	0.00	0.00	0	0	0.00	0.00
NJ	204.95	116.81	88.14	2.31	14,713,412	32,842,874	3.70	160,248
NM	7.96	7.96	0.00	0.38	0	667,668	0.32	83,878
NV	16.50	16.50	0.00	0.55	0	3,707,878	1.22	224,720
NY	1621.69	1335.38	285.81	8.30	88,465,504	278,064,736	14.24	171,466
OH	265.66	136.92	128.74	2.28	16,026,506	37,563,144	3.22	141,396
OK	11.67	7.67	4.00	0.30	1,245,942	924,901	0.23	79,255
OR	31.62	19.62	12.00	0.76	979,291	5,601,628	1.34	177,155

© 2021 Chen C et al. *JAMA Network Open.*

PA	378.99	238.06	140.93	2.96	34,456,596	61,712,368	4.82	162,834
PR	3.84	3.84	0.00	0.12	0	159,245	0.05	41,470
RI	12.10	2.21	9.89	1.14	1,480,888	2,100,010	1.98	173,555
SC	17.19	17.19	0.00	0.34	1,472,536	2,336,631	0.46	135,930
SD	0.00	0.00	0.00	0.00	0	0	0.00	0.00
TN	39.08	39.08	0.00	0.58	3,048,685	6,221,885	0.92	159,209
TX	121.96	61.53	60.43	0.43	5,736,677	12,578,480	0.44	103,136
UT	21.82	13.25	8.57	0.69	1,005,227	2,696,525	0.86	123,580
VA	47.78	26.09	21.69	0.56	1,792,805	8,428,651	0.99	176,405
VT	8.21	4.00	4.21	1.31	448,433	1,254,005	2.01	152,741
WA	47.96	35.96	12.00	0.64	2,290,919	8,930,579	1.19	186,209
WI	30.49	20.11	10.38	0.53	2,117,746	3,812,576	0.66	125,043
WV	8.97	8.97	0.00	0.50	855,009	1,263,096	0.70	140,813
WY	0.00	0.00	0.00	0.00	0	0	0.00	0.00

© 2021 Chen C et al. *JAMA Network Open.*
